# Opposite monosynaptic scaling of BLP–vCA1 inputs governs hopefulness- and helplessness-modulated spatial learning and memory

**DOI:** 10.1038/ncomms11935

**Published:** 2016-07-14

**Authors:** Ying Yang, Zhi-Hao Wang, Sen Jin, Di Gao, Nan Liu, Shan-Ping Chen, Sinan Zhang, Qing Liu, Enjie Liu, Xin Wang, Xiao Liang, Pengfei Wei, Xiaoguang Li, Yin Li, Chenyu Yue, Hong-lian Li, Ya-Li Wang, Qun Wang, Dan Ke, Qingguo Xie, Fuqiang Xu, Liping Wang, Jian-Zhi Wang

**Affiliations:** 1 Department of Pathophysiology, School of Basic Medicine and the Collaborative Innovation Center for Brain Science, Key Laboratory of Ministry of Education of China for Neurological Disorders, Tongji Medical College, Huazhong University of Science and Technology, Wuhan 430030, China; 2 Shenzhen Key Lab of Neuropsychiatric Modulation and Collaborative Innovation Center for Brain Science, CAS Center for Excellence in Brain Science, Shenzhen Institutes of Advanced Technology, Chinese Academy of Sciences, Shenzhen 518055, China; 3 Wuhan Institute of Physics and Mathematics, CAS Center for Excellence in Brain Science, Chinese Academy of Sciences, Wuhan 430071, China; 4 School of Life Science and Technology, Huazhong University of Science and Technology, Wuhan 430074, China; 5 Co-innovation Center of Neuroregeneration, Nantong 226000, China

## Abstract

Different emotional states lead to distinct behavioural consequences even when faced with the same challenging events. Emotions affect learning and memory capacities, but the underlying neurobiological mechanisms remain elusive. Here we establish models of learned helplessness (LHL) and learned hopefulness (LHF) by exposing animals to inescapable foot shocks or with anticipated avoidance trainings. The LHF animals show spatial memory potentiation with excitatory monosynaptic upscaling between posterior basolateral amygdale (BLP) and ventral hippocampal CA1 (vCA1), whereas the LHL show memory deficits with an attenuated BLP–vCA1 connection. Optogenetic disruption of BLP–vCA1 inputs abolishes the effects of LHF and impairs synaptic plasticity. By contrast, targeted BLP–vCA1 stimulation rescues the LHL-induced memory deficits and mimics the effects of LHF. BLP–vCA1 stimulation increases synaptic transmission and dendritic plasticity with the upregulation of CREB and intrasynaptic AMPA receptors in CA1. These findings indicate that opposite excitatory monosynaptic scaling of BLP–vCA1 controls LHF- and LHL-modulated spatial memory, revealing circuit-specific mechanisms linking emotions to memory.

## Abstract (web summary)

How emotions affect memory is an open question. Here the authors establish learnt hopeful and learnt helpless mouse models, and find that posterior basolateral amygdala to ventral hippocampal CA1 monosynaptic glutamatergic inputs link emotions to spatial memory performance.

## Graphical abstract

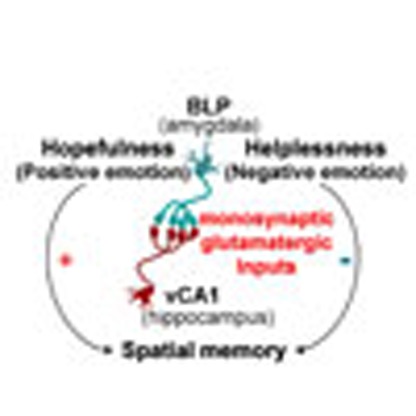

Emotions, categorized into positive and negative along its valence^1^, colour our lives and drive cognitive behaviours in pathophysiological conditions. When facing the challenges, optimism or being positive can motivate adaptive behaviour in the present towards a future goal and it is beneficial to health^2^,^3^. In contrast, a pessimistic view is correlated with the severity of depression^4^, the latter is associated with unrealistic negative predictions of future life events^5^. The current emotion-related laboratory studies are mainly focused on the negative aspects using ‘learned helplessness' (LHL) or depression as model^6^,^7^, and there is a lack of animal model to mimic the positive motivation during stressful experience that may be denominated as ‘learned hopefulness' (LHF).

There are extensive literatures suggesting an association between negative emotion and cognitive impairment. For instance, depression is commonly seen in patients with dementia^8^, such as Alzheimer disease^9^, the latter is characterized by spatial memory deficits^10^. The laboratory animal studies also show that depression can impair memory^11^,^12^. However, the neuronal circuit linking depression with spatial memory impairment is unclear.

The basolateral amygdala (BL) has long been associated with emotion and motivation, playing an essential role in processing both positive^13^,^14^ and negative^15^,^16^,^17^ emotion-associated events, while the hippocampus is essential in the formation of spatial learning and memory^18^,^19^,^20^. BL has neuronal fibres directly projecting to hippocampus^21^, and a monosynaptic connection between anterior BL and ventral hippocampal CA1 (vCA1) has been identified most recently to modulate anxiety-related behaviours^22^. Electrophysiological data show that amygdala regulates long-term potentiation of dorsal hippocampus^23^,^24^, while amygdala lesion by exposure to *N*-methyl-D-aspartate (NMDA) blocks the spatial memory-modulating effect of glucocorticoid in rat hippocampus^25^. On the basis of these studies, we hypothesize that the amygdala–hippocampus interaction may link particular emotion with the hippocampus-dependent spatial memory.

To test this hypothesis, we first established animal models of LHL and LHF. We find that LHF mice show a remarkably increased motivation to escape the aversive foot shocks with the potentiation of spatial learning and memory, while the LHL show despair behaviour with memory deficits. We also discover that the excitatory monosynaptic connection between posterior BL (BLP) and vCA1 is most prominent in physiological conditions, and opposite scaling of the BLP–vCA1 excitatory monosynaptic input governs the LHF- and LHL-modified spatial learning and memory.

## Results

### Opposite influence of LHL and LHF on spatial memory

**Figure 1:**
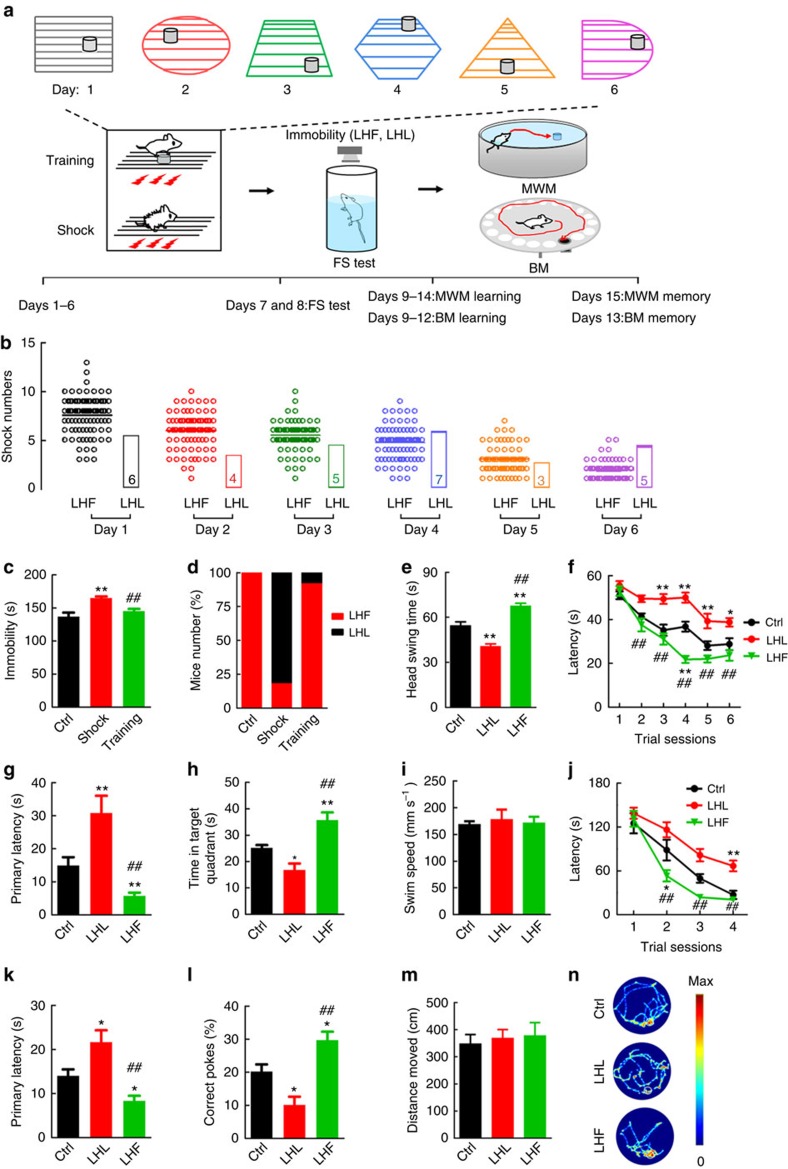
Opposite influence of LHL and LHF on spatial memory in mice. (**a**) Experimental protocol. Mice were exposed to inescapable foot shocks or with simultaneous avoidance training for 6 days in different contextures, and then the learned helplessness (LHL) or learned helpfulness (LHF) was evaluated by forced swimming (FS) and spatial learning and memory were tested by Morris water maze (MWM; Ctrl, *n*=12; LHL, *n*=10; LHF, *n*=10) or Barns maze (BM; Ctrl, *n*=9; LHL, *n*=15; LHF, *n*=12). The Ctrl was exposed to the contextures without shock or training. (**b**) Number of shocks received during each training trial and mean number of shocks (rectangles, 30 times in total) was delivered randomly each day to the simply shocked group. (**c**,**d**) The immobility time in FS was videotaped and the LHL was defined as the immobility time >2 s.d. of the Ctrl (one-way analysis of variance (ANOVA) and Tukey's multiple comparisons test). (**e**) Head swings were recorded during FS, which can distinguish LHF from the Ctrl (one-way ANOVA and Bonferroni's multiple comparison test). (**f**–**h**) MWM data show spatial learning (**f**) and memory (**g**, **h**) deficits in the LHL group and the potentiation in the LHF (two-way ANOVA and Bonferroni's *post hoc* test). (**j**–**l**) BM data show spatial learning (**j**) and memory (**k**,**l**) deficits in the LHL group and the potentiation in the LHF (two-way ANOVA, Bonferroni's *post hoc* test). (**i**,**m**) Swimming speed in MWM test and the distance moved in BM test. (**n**) Representative searching paths during probe trial on BM. Data were presented as mean±s.e.m. *P<0.05, **P<0.01 versus Ctrl; ##*P*<0.01 versus LHL. Ctrl, control.

To explore whether different motivational states affect spatial learning and memory, we exposed the caged mice to helplessly inescapable foot shocks or to the same foot shocks but with avoidance training for 6 consecutive days, and then spatial learning and memory were measured by Morris water maze (MWM; Fig. 1a). To ensure the platform preference and avoid any gradual loss of the escape motivation from environmental familiarity, we increased the difficulty of the avoidance training task by moving the escape platform under different contextures everyday (Fig. 1a). Observation of the videotaped trials indicated that all the trained mice learned to use the electrically isolated platform as a refuge to stay away from the shocks, therefore these mice received decreasing number of shocks during the 6 days training (Fig. 1b). Same amount of foot shocks (30 in total) was delivered in random numbers each day to the inescapable group (Fig. 1b), and the mean cotextural exposure time of the trained group in each day (Supplementary Fig. 1) was evenly given to the inescapable group. The control group (Ctrl) was exposed to the contextures without shock or training. Next, forced swimming (FS) test was used to evaluate ‘behavioural despair' of all the groups on the assumption that the animal has given up HOPE of escaping^26^. About 82% of the helplessly shocked mice lost motivation to escape, whereas ∼92% of the trained mice still had increased escape motivations (Fig. 1c,d). These mice were selected as LHL and LHF, respectively, and used for the related studies. We also noticed that measuring the head swing was more sensitive than the immobility in distinguishing the LHF from the Ctrl (Fig. 1e).

In the following MWM-learning test, the LHF mice learned much faster to find the hidden platform than the Ctrl group, whereas learning deficit was shown in the LHL (Fig. 1f). During the memory test on day 7 by removing the platform, the LHF group shows shorter latency to reach the target quadrant than the Ctrl and LHL groups, while memory deficit was shown in the LHL group versus the Ctrl (Fig. 1g,h). The mice did not show motor dysfunction evaluated by swimming speed (Fig. 1i).

Since MWM test *per se* may induce stress, we then used Barnes maze (BM), a dry-land maze test for hippocampus-dependent spatial reference memory^27^. To exclude the potential perturbations of water maze training on the behaviour in the following BM test, we employed an independent set of mice for the BM studies (Fig. 1a). Again, the LHF group showed faster escape than the Ctrl during 4-day learning trials, whereas learning deficit was seen in the LHL group (Fig. 1j). During the probe trial on day 5, the LHF mice show a significantly shorter latency to reach the target hole with more correct pokes than the LHL and Ctrl groups (Fig. 1k,l,n). No motor dysfunction was detected evidenced by the distance moved in BM (Fig. 1m). The foot shocks without training for 6 days induced similar despair behaviour and memory deficits in the absence or presence of the conducting platform, which excludes the influence of the platform *per se* (Supplementary Fig. 2). Since some of our experiments have to be performed in rats, we tested whether the effects of LHL or LHF on spatial cognitions were also applied in rats. Similar results were observed except that the rats learnt much faster in locating the escapable platform than the mice in MWM (Supplementary Fig. 3). These data together demonstrate that the helplessly repeated foot shocks impair spatial learning and memory, while the escapable training can shift the detrimental effect of the aversive foot shocks into the beneficial.

### Opposite influence of LHL and LHF on BLP–vCA1 circuit

**Figure 2:**
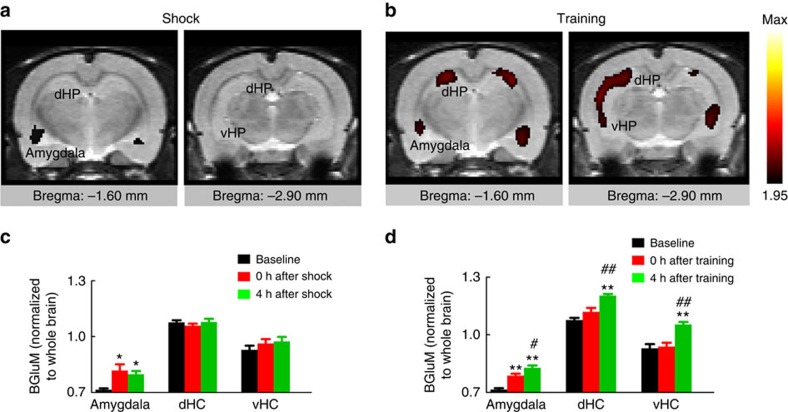
Activity patterns of amygdala and hippocampus in LHF and LHL rats. (**a**,**b**) The representative PET images co-registered to the Schweinhardt MRI template show brain glucose metabolism (BGluM) analysed by Statistical Parametric Mapping (SPM8), normalized to the total brain activity. Red, 0 and 4 h post stimulation>baseline (excitation); *P*<0.05; Ke>100; T>1.95. The baselines were measured 1 week before foot shocks or with avoidance training, and the activity at the indicated brain regions was calculated by normalized to the total brain activity. (**c**) Amygdala activation was detected at 0 and 4 h in the shocked group (*n*=6 per group, one-way analysis of variance (ANOVA), Tukey's multiple comparisons test, *P<0.05 versus baseline). (**d**) A time-dependent activation from amygdala to dorsal and ventral hippocampus (dHC and vHC) was shown in the trained group (*n*=6 per group, one-way ANOVA, Tukey's multiple comparisons test, **P<0.01 versus baseline, #*P*<0.05, ##*P*<0.01 versus 0 h). Data were presented as mean±s.e.m.

To explore the brain regions involved in LHF and LHL mice, we first scanned neural activity of the brains by ^18^F-fluorodeoxyglucose positron emission tomography (PET). Among various brain regions (Supplementary Fig. 4), we observed a sequential activation from amygdala (0 h) to hippocampus (4 h) in the trained mice, but not in the helplessly shocked group (Fig. 2a–d). Neural activation in amygdala and hippocampus was also detected by c-fos staining (Supplementary Fig. 5). Considerable evidence suggests that amygdala plays a crucial role in processing the appetitive or aversive emotion-associated events^13^,^14^,^15^,^16^,^17^, while the hippocampus is essential in spatial memory^18^,^19^,^28^,^29^. These data suggest that the LHF and LHL may oppositely modify amygdala to hippocampus input and thus influence the spatial cognitive functions.

**Figure 3:**
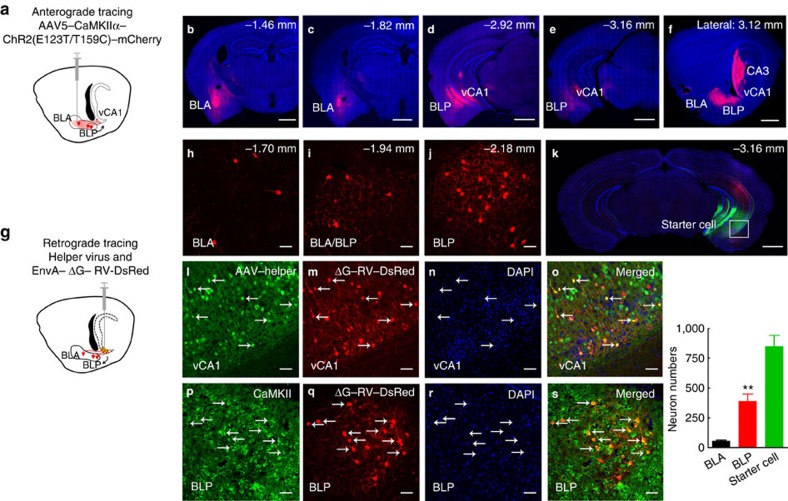
Identification of BLP–vCA1 structural connection in naive mice. (**a**–**f**) Anterograde tracing in C57BL/6 mice: AAV5–CaMKIIα–hChR2(E123T/T159C)–mCherry (red) was injected into the basolateral amygdala nucleus (BL) (**a**); after 6 weeks, the mCherry was predominantly shown in ventral hippocampal CA1 (vCA1) but not in dorsal CA1 viewed by serial coronal sections (**b**–**e**) and sagittal section (**f**). Scale bar, 1 mm. (**g**–**s**) Retrograde monosynaptic tracing in Thy1-Cre mice: the helper viruses AAV-EF1α-DIO-GT and AAV-EF1α-DIO-RV-G (1:1, 200 nl, green) was injected into vCA1, and then rabies virus EnvA-pseudotyped RV-ΔG-DsRed (red) was also injected into vCA1 (**g**). The BLA and BLP neurons that have monosynaptic connection with vCA1 were traced and quantified by DsRed in Thy1-Cre mice (**h**–**k**), and the BLP excitatory neurons were probed by co-staining of DsRed and anti-CaMKII in Thy1-Cre mice (**p**–**s**). The representative images of starter cells in vCA1 at helper virus injection sites (**l**–**o**). (scale bar, 1 mm (**k**) ;50 μm (**h**–**j**,**l**–**s**)). *n*=5 per group, unpaired *t*-test, **P<0.01 versus BLA. Data were presented as mean±s.e.m.

The BL encodes motivationally relevant events^30^. To map the connection between BL and hippocampus, we used virus-delivered trackers to outline the circuit. By injecting the anterograde tracker (AAV5–CaMKIIα–hChR2(E123T/T159C)–mCherry) into BL (Fig. 3a), we observed robust expression of mCherry in ventral, but not dorsal CA1 neurons (Fig. 3b–f), suggesting excitatory neural projection from BL to vCA1. To further verify the direct monosynaptic input from BL to vCA1, we employed a Cre-dependent retrograde monosynaptic tracing system. The mixture of Cre-dependent helper viruses (AAV-EF1α-DIO-GT and AAV-EF1α-DIO-RV-G, 1:1; green) was injected into vCA1 of Thy1-Cre mice to control initial rabies virus infection. Then, EnvA-pseudotyped RV-ΔG-DsRed (red; mutant rabies virus with glycoprotein G gene deletion) was injected into the same site. The helper virus allows the rabies virus spread one synapse retrogradely (Fig. 3g). We could detect DsRed in BL, and, interestingly, the projection signal was much stronger in posterior (BLP) than the anterior (BLA) part of BL (Fig. 3h–j), suggesting a more predominate monosynaptic connection of BLP–vCA1 than BLA–vCA1 in physiological conditions. Finally, we confirmed the excitatory monosynaptic connection of BLP–vCA1 by co-staining of DsRed and CaMKIIα in BLP (Fig. 3p–s).

**Figure 4:**
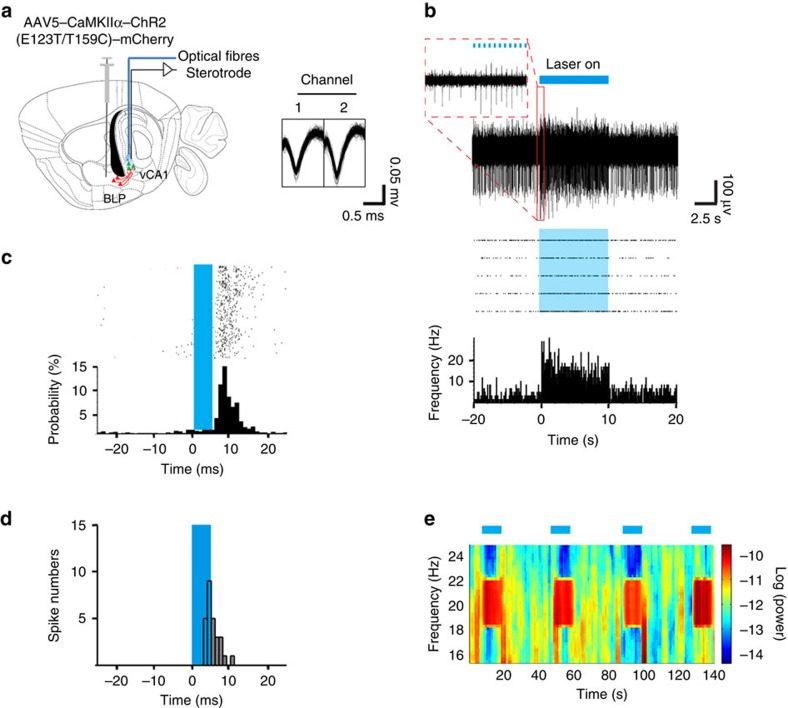
Identification of BLP–vCA1 excitatory monosynaptic connection in naive mice. (**a**) Schematics show BLP injection of AAV5–CaMKIIα–hChR2 (E123T/T159C)–mCherry and vCA1 light stimulation, and the representative traces for spike (*n*=7 cells). (**b**) Top: an example of vCA1 neurons responded to 20-Hz blue light stimulation of BLP–vCA1 terminals, and the insert (red) show 10 light pulses (pulse width=5 ms) of all. Bottom: the representative peristimulus time histogram (PSTH) and raster plots. (**c**) PSTH of the representative vCA1 neurons (aligned by the pulse light onset, blue rectangle) reveals its response probability to the blue light stimulus. (**d**) Histogram of distribution peak response latencies to the onset of light pulses for 27/51 vCA1 neurons (averaged latencies=5.90±1.73 ms). (**e**) The local field potential (LFP) in vCA1 during photostimulation of BLP–vCA1 terminals at 20 Hz.

By *ex vivo* brain slice recording, Felix-Ortiz *et al.* has identified the excitatory monosynaptic connection of BL–vCA1 input^22^. We further employed by *in vivo* extracellular recordings combined with optogenetic stimulation to measure the functional connection between BLP and vCA1 (Fig. 4a). After BLP injection of AAV5–CaMKIIα–hChR2(E123T/T159C)–mCherry, photostimulation at vCA1 (20 Hz, 5-ms light pulse) elicited reliable neuronal spiking with an average firing rate nearly identical to the photostimulation frequency (Fig. 4b). About a half of vCA1 neurons responded to the terminal photostimulation and the peak response latency after light stimulation onset was 5.9 ms (Fig. 4c,d). The light-evoked spike waveforms of vCA1 neurons were shaped with relative broad peak-to-trough width, the characteristic of putative pyramidal neurons (Fig. 4a). The photostimulation could also enhance the power of local field potential at a 20-Hz rhythm (Fig. 4e).

**Figure 5:**
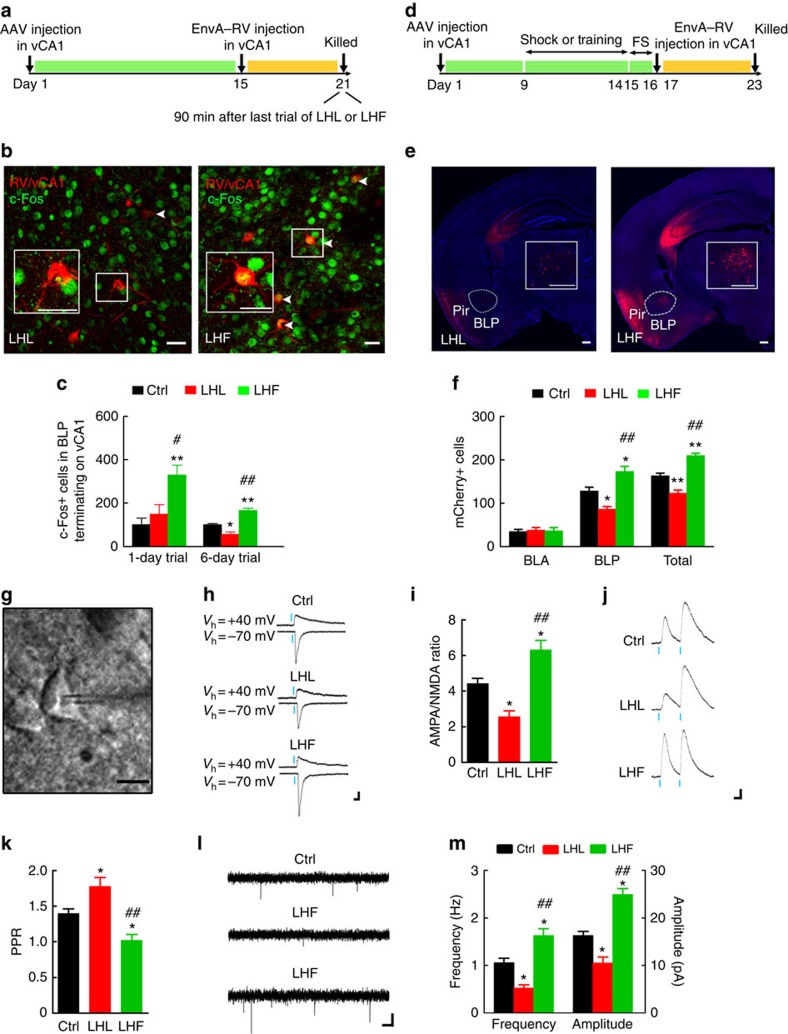
Opposite influence of LHL and LHF on BLP–vCA1 circuit. (**a**) Protocol for measuring the influence of LHF or LHL on the activity of BLP–vCA1 circuit after vCA1 injection of retrograde monosynaptic tracers. (**b**,**c**) The representative co-staining of DsRed+/c-fos+ neurons in BLP (scale bar, 25 μm), and number of c-fos+/DsRed+ neurons in BLP after 1-day trial or 6-day trials (*n*=5 per group, one-way analysis of variance (ANOVA), Tukey's multiple comparisons test, *P<0.05, **P<0.01 versus Ctrl; # *P*<0.05, ##*P*<0.01 versus LHL). (**d**) Protocol for evaluating the structural strength of BLP–vCA1 monosynaptic connection. (**e**,**f**) The representative DsRed images in BLP neurons terminating on vCA1 (scale bar, 250 μm), and number of DsRed+ neurons in BLA (anterior part of amygdala) and BLP (posterior part of amygdala; *n*=5 per group, one-way ANOVA, Tukey's multiple comparisons test, *P<0.05, **P<0.01 versus Ctrl; ## *P*<0.01 versus LHL). (**g**) Infrared-differential interference contrast images of patch pipette tips on a vCA1 pyramidal neuron in a hippocampal slice (scale bar, 10 μm). (**h**,**i**) Representative EPSC_NMDA_ and EPSC_AMPA_ traces recorded from vCA1 pyramidal neuron from different groups and the quantification of EPSC_AMPA_/EPSC_NMDA_ ratio (*n*=5–6 mice per group, one-way ANOVA, Tukey's multiple comparisons test, *P<0.05 versus Ctrl; ## *P*<0.01 versus LHL). Scale bars, 50 pA and 50 ms. (**j**, **k**) EPSP evoked in vCA1 pyramidal neuron by paired photostimuli (50-ms interpulse interval) and the quantification of PPR in slices (*n*=7 mice per group, one-way ANOVA, Tukey's multiple comparisons test,*P<0.05 versus Ctrl; ## *P*<0.01 versus LHL). Scale bars, 1 mV and 20 ms. (**l**,**m**) Representative mEPSC traces recorded from vCA1 pyramidal neurons, and the quantification of mEPSC frequency and amplitude (*n*=4–6 mice per group, one-way ANOVA, Tukey's multiple comparisons test, *P<0.05 versus Ctrl; ##*P*<0.01 versus LHL). Scale bars, 20 pA and 200 ms. Data were presented as mean±s.e.m.

Then, we studied whether and how the BLP–vCA1 inputs are modified in LHL and LHF models. Tamoxifen-induced CaMKIIα-CreERT2 mice were exposed to inescapable foot shocks or shocks with training for 1-day trial or for 6-day trials after (Fig. 5a) or before (Fig. 5d) vCA1 injection of the retrograde monosynaptic tracers. Compared with the Ctrl, the number of c-fos- and DsRed-positive neurons (yellow) in BLP increased in the trained group but no significant change was seen in the simply shocked group when 1-day trial of shocks was delivered; however, remarkable decrease of c-fos+-DsRed+ neurons was shown in the simply shocked group after 6 days of treatment (Fig. 5b,c). These data indicate that short-term training seems enough to activate BLP–vCA1 inputs, but only repeated aversive stresses impair the circuit. Furthermore, the number of DsRed+ cells increased remarkably in BLP but not in BLA of LHF group, whereas it decreased in the LHL group (Fig. 5e, f), which confirms an opposite modulation of the BLP–vCA1 structural strength by LHF and LHL.

To verify the functional strength of BLP–vCA1 inputs modified by LHF and LHL, we injected into BLP with AAV9–CaMKIIα–hChR2(E123T/T159C)–EYFP and did the *ex vivo* electrophysiological recording on acute brain slices at vCA1. The ratio of the light-evoked EPSC_AMPA_/EPSC_NMDA_ significantly increased in LHF mice and decreased in the LHL when compare with the Ctrl (Fig. 5h,i), indicating an opposite modification of BLP–vCA1 functional strength by LHF and LHL on basal condition. To measure the presynaptic changes, we employed a paired-pulse stimulation protocol using light-to-evoke transmission. The paired-pulse ratio (PPR) decreased in the LHF group and increased in the LHL (Fig. 5j,k), which corroborated the changes in miniature excitatory postsynaptic current (mEPSC) frequency and amplitude (Fig. 5l,m). These results indicate that both presynaptic release and postsynaptic processing are involved in the modified BLP–vCA1 input by LHF and LHL.

We also measured on the acute brain slices the total synaptic alterations onto vCA1 after LHF and LHL with *in vivo* photostimulations by electrophysiological recording. The basal synaptic transmission was enhanced in the LHF group over the Ctrl, while blockage of BLP–vCA1 input abolished the effects (Supplementary Fig. 6a,b). The basal synaptic transmission was impaired in the LHL group, while BLP–vCA1 photostimulation rescued the synaptic transmission (Supplementary Fig. 6c,d). The training significantly increased the slope of frequency of excitatory postsynaptic potential (fEPSP) induced by a single train of 100-Hz stimulation (1-s duration) and this potentiation lasted for over 2 h (Supplementary Fig. 6i), suggesting induction of late-LTP, whereas LTP impairment induced by three trains of 100 Hz stimulation was shown in the LHL (Supplementary Fig. 6j). Using a paired-pulse protocol to determine the PPR of fEPSP at CA3–CA1 microcircuit, no significant difference was found among groups (Supplementary Fig. 6e–h). These data further verify that the LHF and LHL can oppositely modify the BLP–vCA1 pathway and change vCA1 synaptic transmission.

### Blocking BLP–vCA1 inputs abolish the LHF-motivated effects

**Figure 6:**
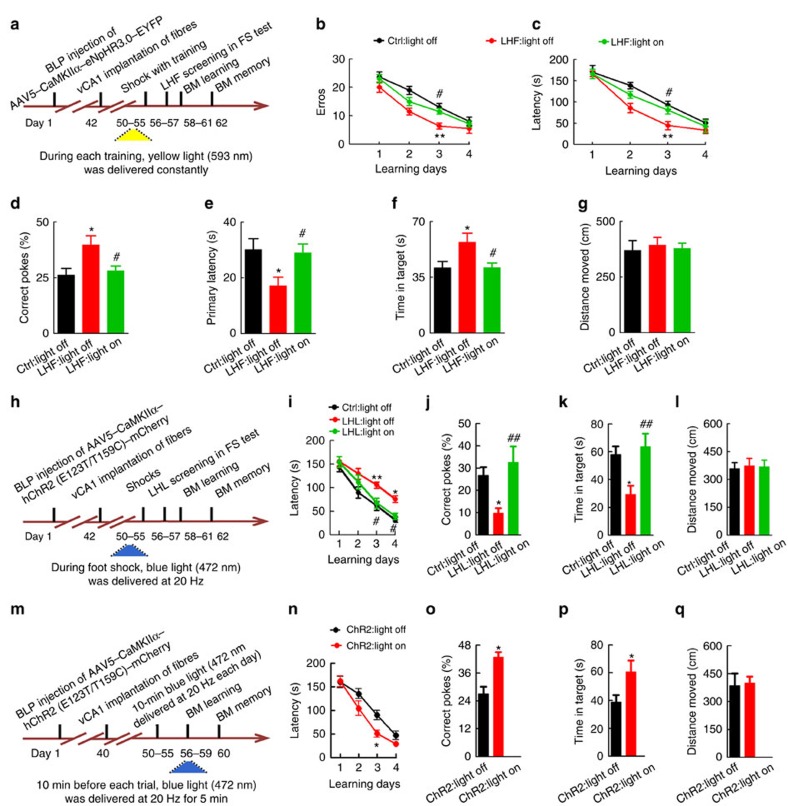
BLP–vCA1 activation is essential for LHF-potentiated spatial memory, and stimulating BLP–vCA1 rescues LHL-induced memory deficit and mimics the effect of LHF. (**a**) Protocol for testing the necessity of BLP–vCA1 activation in facilitating spatial memory of LHF mice. (**b**–**f**) Simultaneous blockage of BLP–vCA1 input by NpHR light on abolishes the LHF-facilitated learning and memory on Barnes maze (BM; *n*=7–8 per group, two-way analysis of variance (ANOVA), Bonferroni's *post hoc* test, *P<0.05, **P<0.01 versus Ctrl; #*P*<0.05 versus light off). (**h**) Protocol for testing the rescue effect of BLP–vCA1 stimulation in LHL mice.(**i**–**k**) Photostimulation of BLP–vCA1 by ChR2 light on improves the LHL-induced learning and memory deficits (*n*=8 per group, one-way ANOVA, Tukey's multiple comparisons test, *P<0.05, **P<0.01 versus Ctrl light off; #*P*<0.05, ##*P*<0.01 versus LHL light off). (**m**) Protocol for testing the sufficiency of BLP–vCA1 stimulation in facilitating spatial memory in naive mice. (**n**–**p**) Photostimulation of BLP–vCA1 input by ChR2 light on for 6 days enhances spatial learning and memory (*n*=8 per group, unpaired *t*-test, *P<0.05, versus ChR2 light off). (**g**,**l**,**q**) Motor function was not affected (*n*=7–8 per group, one-way ANOVA, Tukey's multiple comparisons test or unpaired *t*-test). Data were presented as mean±s.e.m. Ctrl, control.

AAV5–CaMKIIα–eNpHR3.0-EYFP was injected bilaterally into BLP; after 6 weeks, the optical fibres were implanted bilaterally into vCA1. After 7 days of recovery, the mice received avoidance training with simultaneous yellow light on 593 nm in vCA1 for 6 days (Fig. 6a). The targeted blockage of BLP–vCA1 input attenuated the LHF-increased head-swing responses with improved spatial memory (Fig. 6b–f), but without significant change on the immobility in FS (Supplementary Fig. 7a,b). These data suggest that upscaling of BLP–vCA1 input is required for the LHF-facilitated spatial memory, and head-swing response has better sensitivity than the immobility time in identifying the motivation to escape.

### Stimulating BLP–vCA1 rescues LHL-deficit and mimics the LHF

After BLP injection of AAV5–CaMKIIα–hChR2(E123T/T159C)–mCherry and vCA1 implantation of the optical fibres, the mice were exposed to foot-shock trials for 6 days with simultaneous blue light on 472 nm in vCA1, and then the spatial learning and memory were tested in BM (Fig. 6h). The targeted photostimulation of BLP–vCA1 input restored the LHL-induced reduction of head-swing time and immobility (Supplementary Fig. 7c,d) with improvement of learning and memory deficits (Fig. 6i–k). Furthermore, the targeted photostimulation of BLP–vCA1 input for 6 days in naive mice could mimic the facilitating effects of LHF in promoting spatial memory (Fig. 6m–p). No difference in moving distance was observed between light off and light on (Fig. 6g,l,q), indicating no motor dysfunction of the mice.

Photostimulation can induce backpropagating action potentials^31^. To ensure the specificity of BLP onto vCA1 inputs in the light-induced spatial memory potentiation, we combined *in vivo* optogenetic manipulations with the *in vivo* pharmacological manipulations by expressed ChR2 in BLP and implanted a guide cannula in vCA1 (Supplementary Fig. 8a). The saline (Ctrl) or glutamate receptor antagonists, 2,3-Dioxo-6-nitro-1,2,3,4-tetrahydrobenzo [f] quinoxaline-7-sulfonamide (NBQX) and D-2-amino-5-phosphonopentanoate (AP5), were delivered, respectively, through the cannula in vCA1 30 min before illumination. In the Ctrl group, mice replicated the light-enhanced spatial learning and memory on BM task, while administration of the glutamate antagonists abolished the memory potentiation (Supplementary Fig. 8b–d). These data confirm that the glutamatergic BLP inputs onto vCA1 are sufficient for potentiating spatial memory.

### LHL or LHF modulates CA1 plasticity via BLP–vCA1 inputs

**Figure 7:**
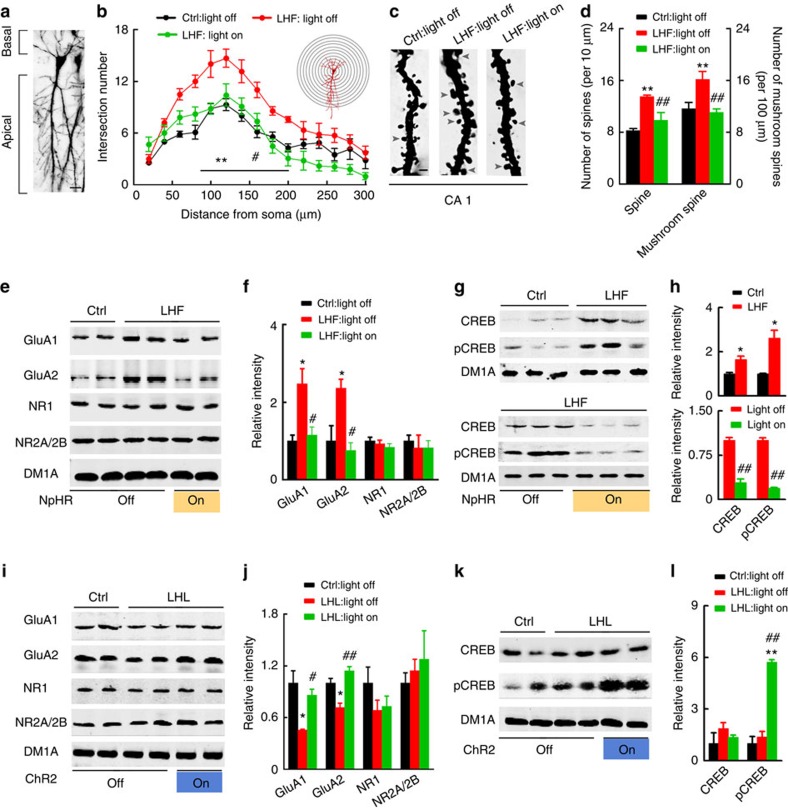
LHF or LHL modulates postsynaptic plasticity via BLP–vCA1 inputs. (**a**)The representative Golgi staining of dendrites in vCA1 (scale bar, 20 μm). (**b**–**d**) LHF increases apical node and spines in vCA1, while blockage of BLP–vCA1 abolishes the increase (at least 20 neurons from six to seven mice per group were analysed by the Sholl, one-way analysis of variance (ANOVA) with Bonferroni's multiple comparisons test, **P<0.01 versus Ctrl; #*P*<0.05, ##*P*<0.01 versus LHF light off; scale bar, 1 μm). (**e**–**h**) LHF increases levels of GluA1/A2 and CREB (pCREB) in CA1, while blockage of BLP–vCA1 abolishes the increase. (**i**,**j**) LHL decreases GluA1/A2 in CA1, while photostimulation of BLP–vCA1 restores the levels. (**k**,**l**) LHL does no change CREB and pCREB in CA1, while photostimulation of BLP–vCA1 increases the pCREB. One-way ANOVA with Tukey's multiple comparisons test, *P<0.05, **P<0.01 versus Ctrl light off; ^#^*P*<0.05, ^##^*P*<0.01 versus LHF light off or LHL light off. Data were presented as mean±s.e.m.

To verify the structural plasticity, we measured the alterations of postsynaptic dendrite complexity, spine density and the morphology in hippocampal CA1 using Golgi-cox staining (Fig. 7a). By the concentric circle (Sholl's) analysis, we found that the neurite arborization (Fig. 7b), spine generation and maturation (Fig. 7c,d) significantly increased in the LHF group compared with the Ctrl, whereas blockage of BLP–vCA1 by NpHR light on abolished the effect (Fig. 7b–d), suggesting an augmented postsynaptic plasticity of BLP–vCA1 circuit in LHF mice. In the LHL group, the spine density in hippocampus decreased (Supplementary Fig. 9). The morphological modification of postsynaptic plasticity was also significant in rats after 6 days of treatment (Supplementary Fig. 10).

In the molecular level, the intrasynaptic GluA1 and GluA2 increased with remarkable upregulation of total and Ser133-phosphorylated CREB (pCREB) in hippocampal CA1 of the LHF group, while simultaneous blockage of BLP–vCA1 by NpHR light on abolished the increase (Fig. 7e–h). The levels of GluA1 and GluA2 decreased in the LHL group with no obvious change of CREB, while simultaneous photostimulation of BLP–vCA1 inputs restored GluA1 and GluA2 with a remarkable increase of pCREB in CA1 subset (Fig. 7i–l). No significant change was observed in NMDA receptor 1 (NR1) and NR2A/2B in LHF or LHL or with the optogenetic manipulations (Fig. 7e,f,i,j). We also observed that targeted photostimulation of BLP–vCA1 inputs for 6 days could mimic the effects of LHF on hippocampal synaptic plasticity with modifications of CREB and intrasynaptic AMPAR in CA1 but not in CA3 and DG subsets (Supplementary Fig. 11). These data suggest that concomitant modifications of CREB and intrasynaptic AMPAR in CA1 subset may serve as molecular modulators to link the BLP-associated emotional stress with the hippocampus-dependent spatial memory.

## Discussion

**Figure 8:**
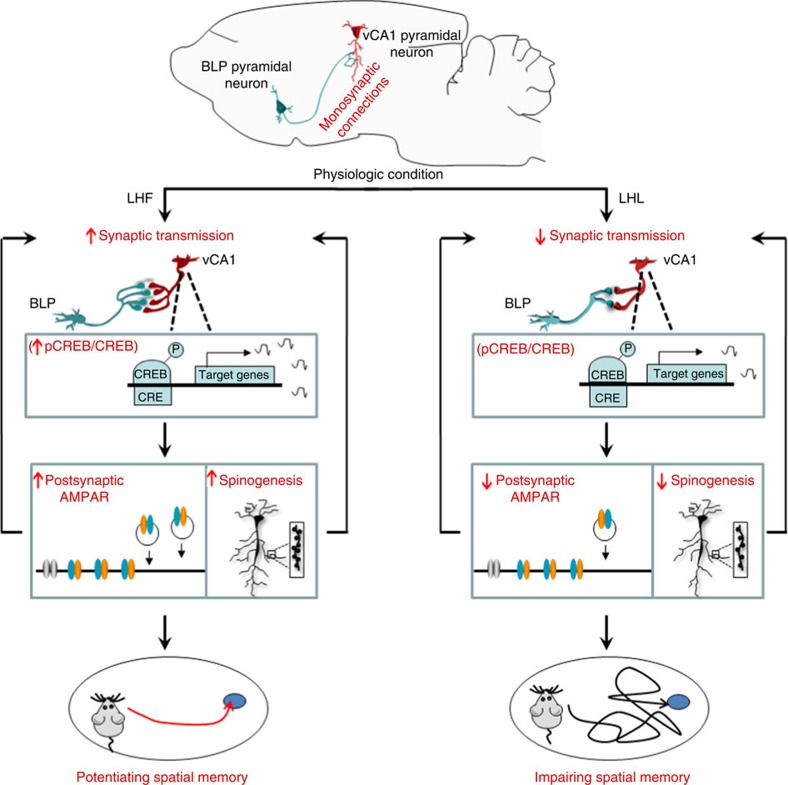
The proposed working model. The excitatory BLP–vCA1 pathway is prominent in physiological condition. The sense of hopefulness reinforces the structural and functional inputs from BLP to vCA1, demonstrated by the activation of pyramidal neurons in vCA1, increases of CREB-dependent postsynaptic membrane insertion of AMPAR with more spine formation and maturation in vCA1, which enhances synaptic transmission and the hippocampus-dependent spatial memory. On the contrary, impairment of BLP–vCA1 inputs by the helplessness reduces postsynaptic AMPAR, then inhibits hippocampal synaptic transmission and impairs the spatial cognitive functions.

How emotions affect cognitive function is a question of broad interests. Here we find that unlike the helpless exposure, the motivated avoidance training enhances spatial memory in murids. The BLP–vCA1 pathway undergoes opposite structural and functional changes following the helpless or hopeful conditioning. Optogenetic manipulations of BLP–vCA1 input reveal causal relationships with valence-affected learning and memory behaviours and the correlated modifications of CREB/AMPA receptors in hippocampal CA1. We propose that the sense of helplessness or hopefulness, possibly originated from the amygdala, can modulate BLP–vCA1 circuit. Specifically, activation of BLP–vCA1 circuit by LHF stimulates pyramidal neuron activity in vCA1 and triggers the CREB-dependent postsynaptic membrane insertion of the AMPAR^32^,^33^, which leads to more spine formation and maturation in vCA1 and thus facilitates hippocampal synaptic transmission, and consequently enhances the hippocampus-dependent spatial memory. On the contrary, impairment of BLP–vCA1 input by the LHL reduces postsynaptic AMPAR, and then inhibits hippocampal synaptic transmission and impairs the spatial cognitive functions (Fig. 8). Our findings reveal a novel neural circuit and the molecular mechanisms that link hopeful or helpless emotion with the altered spatial cognitions, which provides theory evidence for the importance to be hopeful when experiencing aversive stressors. Furthermore, since the amygdala^34^ and hippocampus^10^,^35^ impairments are commonly seen in Alzheimer disease, in which simultaneous psychiatric symptoms and spatial memory deficits are often the early clinical manifestations^9^,^10^,^35^, our findings shed light on this disorder by providing new target for deep brain stimulation, which has recently caught attention in treatment of neurological disorders^36^.

A key component for studying the emotion–cognition interaction is the animal model. Although many social psychological studies imply that the learned optimism is beneficial to health^2^,^3^, it is not reported how this positive motivation when facing on aversive stresses affect memory. One of the major hindrances to this situation is the lack of animal model for learned optimism or LHF. By exposing the animals into repeated foot shocks with the anticipated platform avoidance training for 6 days, we found that ∼92% of the animals tended to escape the aversive stimuli actively and did not show helpless, evaluated by FS test^26^. The procedure has good reproducibility and simple handling; therefore, it may serve as an ideal paradigm for learnt hopefulness. Interestingly, we did not see significant difference between LHF and the Ctrl by measuring the immobility during FS although the spatial memory was significantly improved in the LHF group. However, when the head swing was used to measure the motivation to escape during FS^37^, the LHF could be distinguished from the Ctrl. These data perfectly link the motivation with the improved spatial memory in our LHF paradigm. On the other hand, ∼50% of the LHL mice produced by tail shocks show despair behaviour^38^. By our repeated foot-shock paradigm, ∼82% of the animals show LHL.

The BL, including basal and lateral nuclei of the amygdala, connects with many brain regions^39^,^40^ and encodes motivationally relevant events, such as fear^30^,^41^, anxiety^22^,^42^ and the cue-induced award^13^,^30^. The anatomic link between BL and hippocampus was reported previously using localized injections of the anterograde tracer phaseolus vulgaris-leucoagglutinin (PHA-L) into specific subdivisions of the amygdala^21^. Recently, monosynaptic connection between anterior basolateral amygdale and vCA1 has been identified by anterograde tracing^22^. The conventional anterograde and retrograde neuronal tracers can locate the neurons projecting to or from particular brain regions, but these methods create ambiguity about whether the cells are directly or indirectly connected^43^. To improve this, we developed Cre-dependent helper virus to control initial rabies virus infection in vCA1 and subsequent retrograde monosynaptic spreading. Using this retrograde monosynaptic tracing, we find that BLP–vCA1 connection is more prominent than that of BLA–vCA1 *in vivo*, which offers a precise supplement for amygdale outputs in physiological condition. Though weaker connection of BLA–vCA1 was also detected by our retrograde tracing, the involvement of BLA–vCA1 pathway in LHF or LHL paradigm deserves further investigation.

We chose to study the BLP–vCA1 pathway mainly based on the anterograde tracing data that show prominent BLP projection to vCA1 but not to the dorsal. Although the ventral hippocampus seems more concerned with emotions, such as anxiety and fear^44^, studies also show its involvement in learning and memory. For instance, the ventral hippocampus-involved ‘simplified learning' accelerates subsequent maze learning and reduces its requirement for NMDA-mediated plasticity^45^. In MWM, ventral hippocampus mediates early task-specific goal-oriented searching^20^. Furthermore, ventral hippocampal–prefrontal input encodes critical location information during spatial working memory tasks^19^. Our current study also provides strong evidence supporting the role of BLP–vCA1 inputs in modulating spatial memory. Nonetheless, since alteration of other brain regions, such as CA3 and dCA1, was also detected by c-fos staining or PET scan, thus further studies may clarify whether vCA1 *per se* regulates spatial memory or it does so through interacting with other brain regions in LHF and LHL paradigms.

It is well recognized that the cAMP–CREB pathway regulates learning and memory^46^,^47^, but its role in emotion-associated spatial memory remains unknown. Our data show that the enhanced basal synaptic transmission is coupled with an increased expression of intrasynaptic AMPA receptors and CREB upregulation in CA1 but not the other subsets of the hippocampus after targeted BLP–vCA1 photostimulation. Furthermore, the effect on AMPAR is subunit specific as indicated by a lack of any significant changes in NR1 and NR2A/2B subunits. Our data also show that the change of CREB in hippocampal CA1 was most significant in the trained and the BLP–vCA1 stimulated mice, which is in good agreement with the observations that upregulation of CREB can upregulate AMPAR^32^,^33^, and increases length, branching and spine density of dendrites in hippocampal neurons^48^. Thus, upregulation of CREB in hippocampal CA1 may underlie the enhanced postsynaptic plasticity induced by BLP–vCA1 upscaling. On the other hand, the CREB was not changed in LHL paradigm, although simultaneous photostimulation of BLP–vCA1 significantly increased pCREB, suggesting that other molecular mechanisms than CREB may be involved in the memory deficits of the LHL mice.

In summary, we have identified a prominent excitatory connection between BLP and vCA1, and we find that opposite scaling of BLP–vCA1 inputs control the LHF- and LHL-associated spatial memory by modifying the synaptic plasticity and CREB/AMPAR in hippocampal CA1 subset.

## Methods

### Animals

Adult male C57BL/6 mice (6–8-week old) were purchased from the Center for Animal Experiment/Animal Biosafety Level-III Laboratory, Wuhan University (Wuhan, China). Thy1-Cre transgenic mouse model was a kind gift of Dr S. Duan (Zhejiang University, Hangzhou, China). CaMKIIα-CreERT2 transgenic mouse model was from the Model Animal Research Center of Nanjing University. Adult male Sprague Dawley rats (60–70-day old, 250–300 g) were from the Experimental Animal Center of Tongji Medical College, Huazhong University of Science and Technology. All animal experiments were approved by the Animal Care and Use Committee of Huazhong, University of Science and Technology, and performed in compliance with the National Institutes of Health Guide for the Care and Use of Laboratory Animals. The animals were housed in groups of three to five per cage with free access to food and water, and were maintained on 12-h light to 12-h dark cycle (lights on at 5:00 PM, off at 5:00 AM) at stable temperature (23–25 °C). The animals were extensively handled (2 min everyday for 7 days) before each behavioural test.

### Learned hopefulness and learned helplessness

The animals were brought into the box for 2 min to acclimate the environment, and then foot shocks (2 s duration, 1.0 mA current intensity) were delivered through copper electrodes augmented with electrode paste by a Precision Regulated Animal Shocker (Coulbourn Instruments) to mice restrained in multishaped acrylic boxes (base area: 200±16 cm^2^) or to rats in 50 × 25 × 25-cm boxes with different contextures. Each box was enclosed in a sound-attenuating chamber.

For the LHF group, an insulating rubber platform (4.5-cm high and 4.8-cm wide for mice, 5-cm high and 8-cm wide for rats) was placed in the box and the animals were trained to climb on it^49^. At the beginning, the animals generally stepped down after 3–10 s stay on the platform, and then the foot shocks were delivered again. When the animals were trained to stay on the platform with four limbs or to hold on to it with at least three limbs for up to 60 s, we stopped training for the day. The procedure was repeated for 6 consecutive days. To ensure the platform preference and avoid any gradual loss of the escape motivation from environmental familiarity, we moved the platform everyday at different contextures.

For the LHL, the mean number of foot shocks received by the LHF (30 times in total) was delivered randomly for 6 days. The mean contextual exposure time of the trained group in each day was evenly given to the inescapable group. In this paradigm, the rubber platform was removed or substituted for a conducting one with the same size and shape, but the animals were not trained to climb the platform. If the animals occasionally touch the platform, they still receive electric shocks. The platform location and the contextures of the boxes were also changed everyday as did in the LHF group. The Ctrl was exposed to the contextures but without foot shocks or training.

### FS test

Mice or rats were placed individually in the transparent plexiglas cylinder (20-cm high and 15-cm diameter) containing fresh water (24–26 °C) to a height of 15 cm. The animals were allowed to adapt to the environment for 5 min before test. Each test was lasted for 6.5 min and the duration of immobility was recorded after the first 0.5 min. The despair behaviour or LHL was defined as that the immobility time was two s.d.'s greater than the Ctrl. Head-swing time was also recorded to estimate escaping motivation during FS^37^.

### MWM test

The maze was located in a room containing several visual cues. The water (24–26 °C) in the tank was made opaque with milk (for mice) or Chinese ink (for rats) to hide the escape platform. A camera, fixed to the ceiling with 1.5 m from the water surface, was connected to a digital tracking device. A computer with the water maze software then processes the tracking information. The animals were kept on outer-room shelves to eliminate directional olfactory and auditory cues, and they were brought to the site 2 h before test. A learning session was consisted of four trials with a 30-s interval, lasting for 6 days. On each trial, the animal started from one of the four quadrants facing the wall of the pool and ended when it climbed the platform. The animals were not allowed to search for the platform >60 s, after which they were guided to the platform. The learning curve was made by the latency that the animals could find the platform. For memory test, the escape latency, platform crossings, time in target quadrant and the swimming paths were recorded at 24 h after the last learning trial by removed the platform.

### BM test

The animals were habituated in the escape box for 3 min at 24 h before test. In the next 4 days of learning trials, three trials per day with a 30-min interval were implemented^27^. The animals were held in a cylindrical chamber in the centre of the maze for 10 s before each trial, and then they were trained to find the target hole within 3 min. Once unable to find the target hole at the end of each trial, they were guided to the target hole and stayed in the escape box for 1 min. In between each trial, 70% ethanol and paper towels soaked with water were used to clean the platform and the escape box. On day 5, the animals were allowed to search freely for 1.5 min with escape box removed. During the learning trials, the latency to the target hole and the number of errors were recorded by video-tracking software. During the probe trial, the latency to the target hole, time spent in the target quadrant, percentage of correct pokes and the distance moved were measured.

### ^18^F-fluorodeoxyglucose positron emission tomography

After intraperitoneal injection of ^18^F-fluorodeoxyglucose (0.6–0.7 mCi, Cardinal Health), the rats were anaesthetized (1.5–2.0% isoflurane) and imaged using R4 PET Tomograph (Concorde CTI, Siemens), which has a transaxial resolution of 2.0 mm full width at half maximum with a field of view of 11.5 cm. The rats were placed in the centre of the field of view and dynamic scans were taken for 30 min (21 frames: 6 frames, 10 s; 3 frames, 20 s; 8 frames, 60 s; 4 frames, 300 s) and then averaged. The images were corrected for photon emission and reconstructed using the OSEM3D/MAP algorithm provided by Concorde CTI. After baseline recording, the rats were treated with foot shocks or with avoidance training for 6 days, immediately after (0 h) or 4 h after foot shocks or avoidance training, PET images were recorded again. PMOD software (v.2.75, PMOD Technologies Ltd) was used to manually draw regions of interest in specific brain region cluster (at given SPM coordinates), and the activity in regions of interest (such as amygdala) was normalized to the whole brain.

### Anterograde tracing

Mice were anaesthetized with chloral hydrate (36 mg per 100 g body weight) and placed in a rodent stereotaxic frame (David Kopf Instruments model 900 series). The craniotomy was made using anterior-posterior (AP) coordinates from bregma (in mm), medo-lateral (ML) coordinates from midline and dorso-ventral (DV) coordinates from the brain surface. For all surgeries, the body temperature of the animals was maintained using a heat lamp. The AAV5–CaMKIIα–hChR2(E123T/T159C)–mCherry^50^ (0.6–0.8 μl, >10^12^ vg ml^−1^) was delivered into the basolateral amygdala nucleus (BL) with coordinates −2.0 AP, ±3.1 ML and −4.4 DV. Six weeks later, the mice were anaesthetized with a lethal dose of chloral hydrate and were transcardially perfused with 0.9% saline followed by ice-cold phosphate buffer containing 4% paraformaldehyde. The expression of mCherry in the amygdala and hippocampus was detected by immunofluorescence imaging with a confocal microscope (LSM 710, ZEISS, Germany).

### Retrograde monosynaptic tracing

The mixed helper virus containing AAV-EF1α-DIO-GT (6.53 × 10^12^ vg ml^−1^ and AAV-EF1α-DIO-RV-G (5.23 × 10^12^ vg ml^−1^; 1:1, 200 nl) were injected into vCA1 of Thy1-Cre or Tamoxifen-induced CaMKIIα-Cre transgenic mice with coordinates of −3.16 AP, −2.95 ML, and −4.15 DV. Two weeks (for CaMKIIα-CreERT2 transgenic mice) or four weeks (for Thy1-Cre transgenic mice) later, rabies virus EnvA-pseudotyped RV-ΔG-DsRed (2 × 10^9^ vg ml^−1^, 200 nl) was injected into the same site of vCA1. About seven days after the injection, mice were anaesthetized with urethane (1 g kg^−1^) and were transcardially perfused, and then the brain slices were prepared for tracing DsRed or co-staining with CaMKII in BLP.

### Free-moving mice optogenetic manipulation and behaviour test

The AAV5–CaMKIIα–eNpHR3.0–EYFP or AAV5–CaMKIIα–hChR2(E123T/T159C)–mCherry (0.6–0.8 μl, >10^12^ vg ml^−1^) was injected bilaterally into BLP (−2.0 AP, ±3.0 ML and −4.7 DV) at a speed of 0.1 μl min^−1^. After 6 weeks, animals were implanted bilaterally with optical fibres (200 μm core, numerical aperture=0.37) held in a ceramic ferrule (Fibers, Shanghai, PRC) in vCA1 (−3.16 AP, ±2.95 ML and −3.6 DV), and the implants were secured to the skull with dental cements. During behavioural tests, the laser was first connected to a patch cord with a pair of FC/PC connectors in each end. This patch cord is connected through an optic fibre rotary joint, which allow free rotation of the fibre, with another patch cord that has a FC/PC connector and a deliver of the laser chronic optic fibres (Doric, Québec, Canada). For optogenetic inhibition of BLP–vCA1 input using eNpHR3.0, 8–10 mW (∼35.35 mW mm^−2^ at the tip of the fibres) of constant yellow light generated by a 100-mW, 593-nm DPSS laser was delivered. For mice using hChR2 (E123T/T159C), 6–8 mW (∼53.03 mW mm^−2^ at the tip of the fibre) of light trains at 20 Hz, 5 ms pulses of blue light generated by a 100-mW, 472-nm DPSS laser was delivered. Laser output was manipulated with a Master-8 pulse stimulator (A.M.P.I., Jerusalem, Israel).

NBQX and AP5 were purchased from Tocris (Tocris, Ellisville, MO) and dissolved in saline (0.9% NaCl) to make glutamate antagonist cocktail (NBQX, 22 mM; AP5, 38 mM). The cocktail (0.5 μl) was infused into the vCA1 at a flow rate of 0.1 μl per min via an internal needle at 30 min before the behavioural assays and optogenetic manipulations.

### Single-unit spike sorting and optogenetic analysis in mice

The AAV5–CaMKIIα–hChR2(E123T/T159C)–mCherry was injected into BLP and optic fibres were implanted in vCA1. A micro-electrode array was inserted in vCA1 with the help of a stepping motor (IVM; Sentifica, Uckfield, USA) at a speed of 1 μm s^−1^. The array consisted of eight stereotrodes made by insulated nichrome wires (OD=17 μm; CFW, California, USA), which were electrochemically modified until the impedance <0.5 MΩ. Data were collected in anaesthetized living mice using OmniPlex D Neural Data Acquisition System (Plexon, Dallas, USA). Stable spontaneous spikes of vCA1 pyramidal neurons were detected when 20 Hz with 5 ms width pulsed laser was delivered. Multi-unit spike data were sorted manually in off-line (Plexon Offline Sorter software). The quality of a single unit was evaluated by the autocorrelograms to detect the presence of an absolute refractory period (>1 ms), only unit clusters containing <1% of the spikes with inter-spike interval <1 ms were included. Putative pyramidal neurons were defined by relatively broad spike waveforms (peak to trough >380 μs).

### Electrophysiological recording on brain slices

The brain slices were cut (300 μm) in ice-cold artificial cerebrospinal fluid (ACSF) containing (in mM): NaCl 124; KCl 3.0; MgCl_2_ 1.0; CaCl_2_ 2.0; NaH_2_PO_4_ 1.25; NaHCO_3_ 26; glucose 10; saturated with 95% O_2_, 5% CO_2_ (pH 7.4). Then, slices were incubated at 32 °C for 30 min in ACSF, and allowed to equilibrate to room temperature for >30 min.

For whole-cell patch-clamp experiment, recordings were made from visually identified pyramidal neurons in the pyramidal layer of the vCA1 region after 6–7 weeks of AAV9–CaMKIIα–hChR2(E123T/T159C)–EYFP infection in BLP. For current-clamp experiments, borosilicate glass capillaries were pulled on a P-97 puller (Sutter Instrument, Novato, CA) and the recording electrodes (7–9 MΩ) were filled with (in mM): 135 potassium gluconate, 4 KCl, 2 NaCl, 10 HEPES, 4 EGTA, 4 MgATP, 5NaGTP, 280 mOsm kg^−1^, pH adjusted to 7.4 with KOH). For voltage-clamp experiments, borosilicate glass capillaries were pulled on a P-97 puller (Sutter Instrument, Novato, CA) and the recording electrodes (7–9 MΩ) were filled with (in mM): 120 cesium methansulphonate, 20 HEPES, 0.4 EGTA, 2.8 NaCl, 5 tetraethylammonium chloride, 2.5 MgATP, 0.25 NaGTP (pH 7.4, 285 mOsm kg^−1^). All recordings were made using a Multiclamp 700B amplifier (Molecular Devices, Sunnyvale, CA). Analogue signals were low-pass filtered at 1 kHz and digitized at 10 kHz using a Digidata 1440 and pClamp9 software (Molecular Devices, Sunnyvale, CA). ACSF and drugs were applied to the slice via a peristaltic pump (Minipuls3; Gilson, Middleton, WI) at 2 ml min^−1^. Off-line analysis was performed using Clampfit software (Molecular Devices, Sunnyvale, CA).

For ChR2 excitation, square pulses of blue light (472 nm, 5 ms in duration) were delivered through × 40 water-immersion objective of a Nikon FN1 microscope. A 300-W Xenon lamp (Lambda DG5, Sutter, US) served as a light source. The light power at the microscope objectives was 2–3 mW mm^−2^. To set light pulse and train duration, the DG5 wavelength switcher were controlled by Digidata 1440, which was triggered by the corresponding Clamp10 software (Molecular Devices, US).

For extracellular recording, individual slices from Ctrl, LHL and LHF with *in vivo* optogenetic manipulation were laid down over an 8 × 8 array of planar microelectrodes, each 50 × 50 μm in size, with an interpolar distance of 150 μm (MED-P515A; Alpha MED Sciences, Kadoma, Japan) and kept submerged in ACSF (4 ml min^−1^; 30 °C) with a nylon mesh glued to a platinum ring. Voltage signals were acquired using the MED64 System (Alpha MED Sciences). The fEPSPs was obtained by electrically stimulating the CA3 region. Stimulation intensity was adjusted to evoke fEPSP amplitudes that were 30% of maximal size. Long-term potentiation (LTP) was induced by applying one or three train(s) of high-frequency stimulation (100 Hz, 1-s duration at test strength). Paired-pulse facilitation in hippocampal CA1 was evaluated in separate slices under identical conditions as used for LTP. Paired-pulse facilitation was measured by evoking fEPSP with varying interpulse intervals from 50 to 300 ms, and expressed as fEPSP2/fEPSP1.

### Golgi staining

The anaesthetized animals were perfused with 0.9% NaCl containing 0.5% sodium nitrite, followed by 4% formaldehyde, and then perfused with dying solution made of 5% chloral hydrate, 5% potassium dichromate and 4% formaldehyde. The brains were removed and further incubated in the above dying solution for 3 days and transferred to a solution containing 1% silver nitrate for another 3 days in dark. Then, the brains were sliced using a Vibratome (VT1000S; Leica, Germany) at a thickness of 40 μm. The images at CA1 pyramidal neurons were taken by bright-field microscopy (Axioplan 2; Zeiss, Brighton, MI). Images were coded, and the dendritic spines from at least 60 neurons per group were counted in a blind manner using Neurolucida software (MicroBrightField, Williston, VA). For Scholl analysis, at least 10 neurons in vCA1 of hippocampus were used for each group^49^.

### Immunofluorescence staining

Mice were killed 1.5 h after the last trial of shock or with training by a lethal dose of chloral hydrate and were transcardially perfused with 0.9% NaCl followed by 4% paraformaldehyde. The brains were removed and post-fixed in perfusant overnight, and then sectioned using a Leica microtome (30 μm in thickness). Sections were thoroughly washed with PBS-T (PBS containing 0.2% Triton X-100) and preincubated with 5% normal goat serum. Subsequently, the sections were incubated overnight with monoclonal mouse CaMKIIα (1:200; Thermo Scientific, Rockford, IL, USA) or polyclonal rabbit c-fos (1:5,000; F7799, Sigma, MO) in 1% normal goat serum containing 0.2% Triton. After that, the sections underwent three washes in PBS-T, followed by 1-h incubation with the secondary antibody (1:200, A-11029, Invitrogen or A-11034 Invitrogen). The slice then underwent three more washes, followed by cover slipping on microscope slides.

### Western blotting

The sub-brain regions were isolated carefully with a sharp needle-tip (for details, see http://www.ncbi.nlm.nih.gov/pmc/articles/PMC3142893). The synaptosome (P2 fraction) was prepared by gradient sucrose centrifugation. The proteins in total extracts or in P2 fractions were separated by SDS–polyacrylamide gel electrophoresis, and probed with antibodies against CREB (1:500), pCREB (1:500), NR1 (1:1,000), NR2A/2B (1:1,000), GluA1 (1:1,000), GluA2 (1:1,000) and DM1A (1:1,000). The blots were developed with horseradish peroxidase-conjugated secondary antibodies and visualized by enhanced chemiluminescence substrate system (Santa Cruz Biotechnology Inc, Santa Cruz, CA, USA). The protein bands were quantitatively analysed by Kodak Digital Science 1D software (Eastman Kodak Company, New Haven, CT, USA). The blots have been cropped for presentation, and full-size images are presented in Supplementary Fig. 12 and supplementary Fig. 13.

### Statistical analyses

Data were expressed as mean±s.e.m. and analysed using commercial software (GraphPad Prism; GraphPad Software, Inc, La Jolla, CA). The two-way analysis of variance or one-way analysis of variance, or a Student's *t*-test was used to determine the different means among the groups. The level of significance was set at *p*<0.05.

### Data availability

The authors declare that the data supporting the findings of this study are available within the article and its Supplementary Information files or from the authors upon request.

## Additional information

**How to cite this article:** Yang, Y. *et al.* Opposite monosynaptic scaling of BLP–vCA1 inputs governs hopefulness- and helplessness-modulated spatial learning and memory. *Nat. Commun.* 7:11935 doi: 10.1038/ncomms11935 (2016).

## Supplementary Material

Supplementary Information Supplementary Figures 1-13
