# Supplementary material for: Opposite monosynaptic scaling of BLP–vCA1 inputs governs hopefulness- and helplessness-modulated spatial learning and memory
Source: Nat Commun. 2016 Jul 14;7:11935. doi: 10.1038/ncomms11935 (PMC4947155; doi:10.1038/ncomms11935)

Supplementary Figure 1

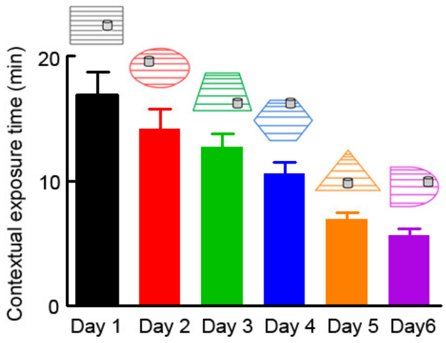

Supplementary Figure 1 Contextual exposure time during avoidance training. Mice were exposed to foot–shocks with avoidance training for 6 consecutive days as shown in **Fig. 1a, b**. The contextual exposure time was recorded every day. The average exposure time for each day is presented as mean ±s.e.m.

Supplementary Figure 2

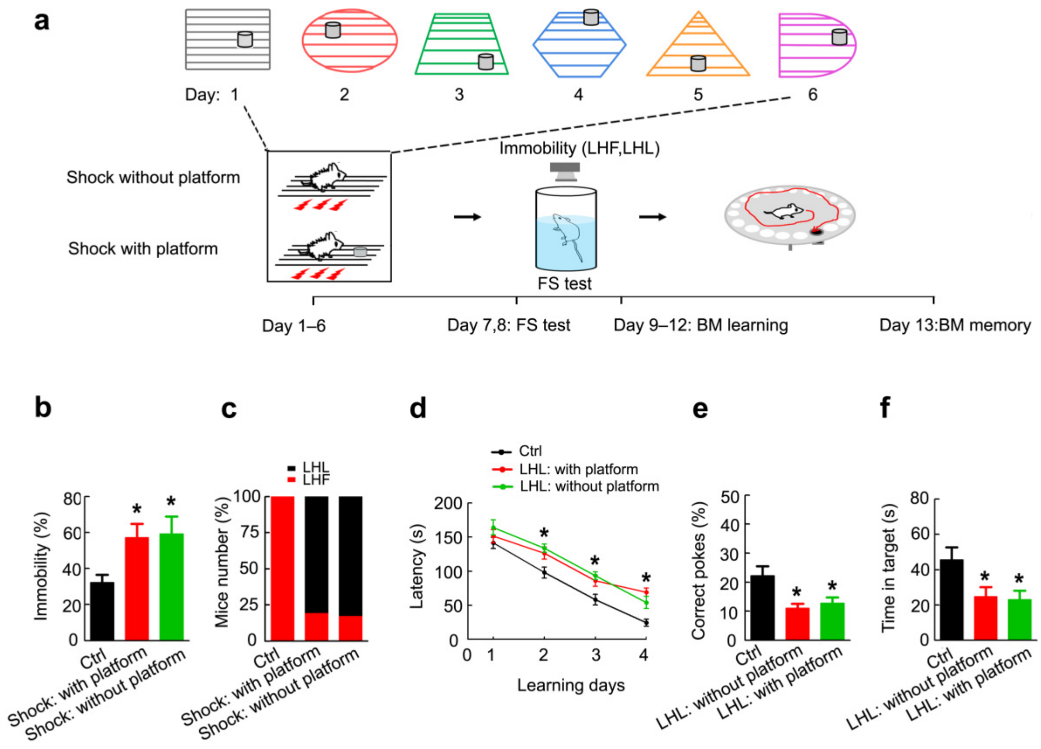

Supplementary Figure 2 Foot–shocks induce helplessness and memory deficit independent of the platform. (**a**) Experimental protocol. The mice were exposed to foot–shocks for 6 days as shown in **Fig. 1a, b** with or without a moveable conducting platform. (**b**, **c**) The immobility during FS were videotaped and analyzed (n=12, Ctrl, n=10, without platform, and n=13, with platform; One–way ANOVA, Tukey'smultiple comparisons test). (**d–f**) Spatial learning (**d**) and memory (**e**, **f**) were measured on BM (n=12, Ctrl, n=8, without platform, and n=10, with platform; Two–way ANOVA, Bonferroni'spost hoc test). Data were presented as mean ±s.e.m. *P<0.05 *versus* Ctrl.

Supplementary Figure 3

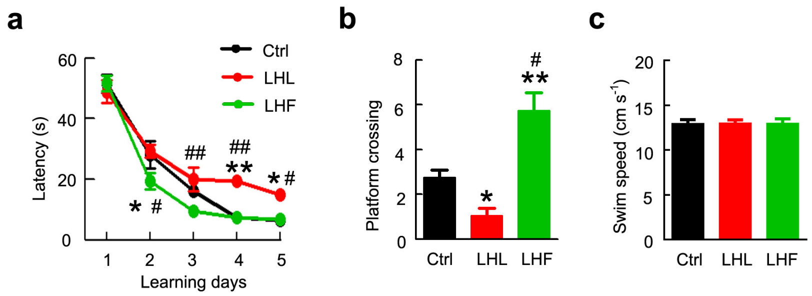

Supplementary Figure 3 Opposite influence of LHL and LHF on spatial memory in rats. (**a**) Rats were treated as indicated in **Fig. 1a, b**. A prolonged latency to reach the target platform on day 4 and 5 during learning trials was shown in LHL group, while enhanced learning capacity on day 2 was observed in the LHF compared with the Ctrl. (**b**) In the memory test given on day 6 by removed the escape platform, the rats in LHL group show reduced crossing in the target quadrant while the LHF group show increased crossing in the target quadrant. (**c**)The swimming speed was not changed. Data were presented as mean ±s.e.m. n=9–10 per group. *P<0.05, **P<0.01 *versus* Ctrl; # P<0.05, ##P<0.01 *versus* LHF (a) or LHL (b).

Supplementary Figure 4

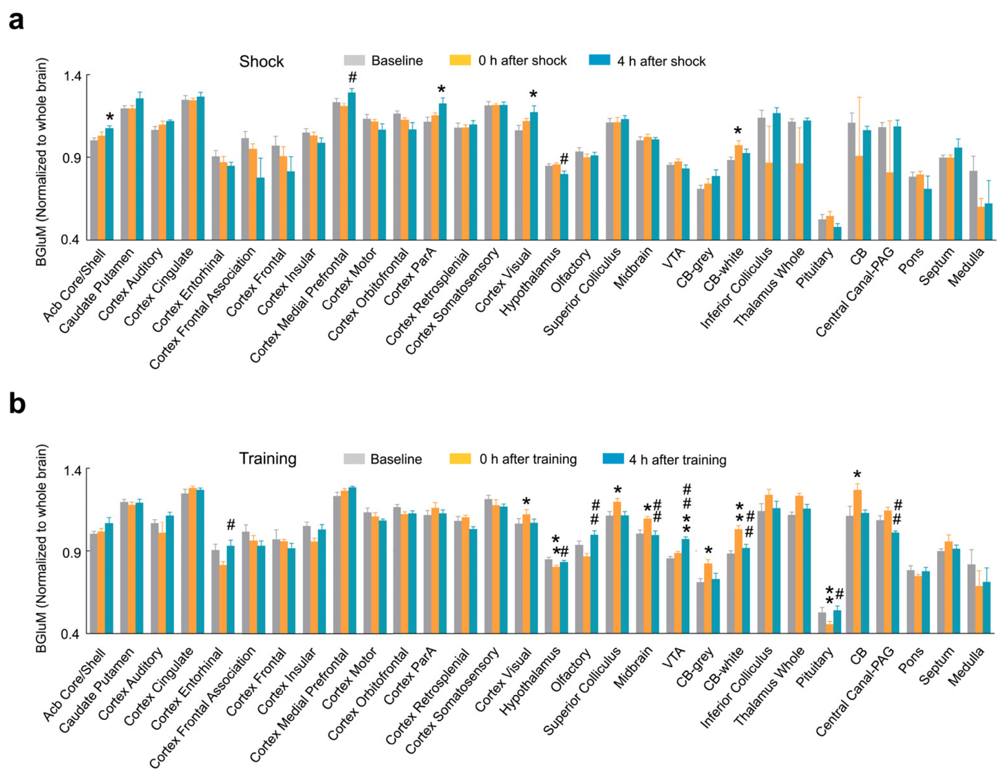

Supplementary Figure 4 Positron emission tomography (PET) analyses in rats. The LHL (**a**) or LHF (**b**) rats were treated as indicated in **Fig. 1a, b**, then the glucose metabolism in brain subregions (BGluM) was measured by PET scan. The data were analyzed by statistical parametric mapping (SPM8) and normalized to the whole brain. Data were presented as mean±s.e.m, n=6–8 per group, One–way ANOVA with Tukey's multiple comparisons test, * $P<0.05$， ** $P<0.01$ *versus* baseline; #$P<0.05$， ##$P<0.01$ *versus* 0 h.

Supplementary Figure 5

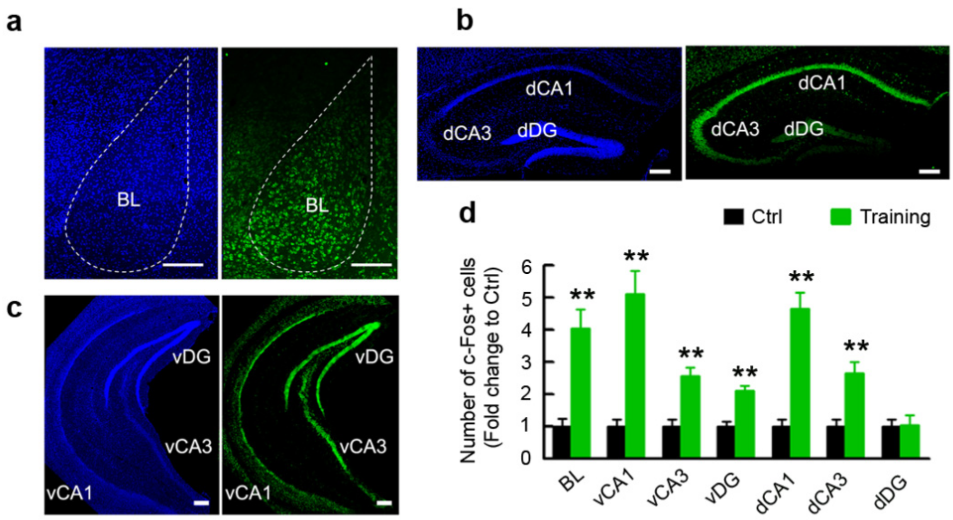

Supplementary Figure 5 The LHF increases c–fos in amygdala and hippocampus. The mice received foot–shocks with avoidance training for 6 days as shown in **Fig. 1a**. (**a–c**) Representative co–staining of c–fos (green) and Hoechst (blue) in basolateral amygdala (**a**) and hippocampus (**b**, **c**). Scale bars, 100 µm. (**d**) Number of c–fos–positive cells increased in BL, vCA1, vCA3, vDG, dCA1 and dCA3 but not in dDG.Data were presented as mean ±s.e.m. n=6 per group, unpaired t test, ***P*<0.01 versus Ctrl.

Supplementary Figure 6

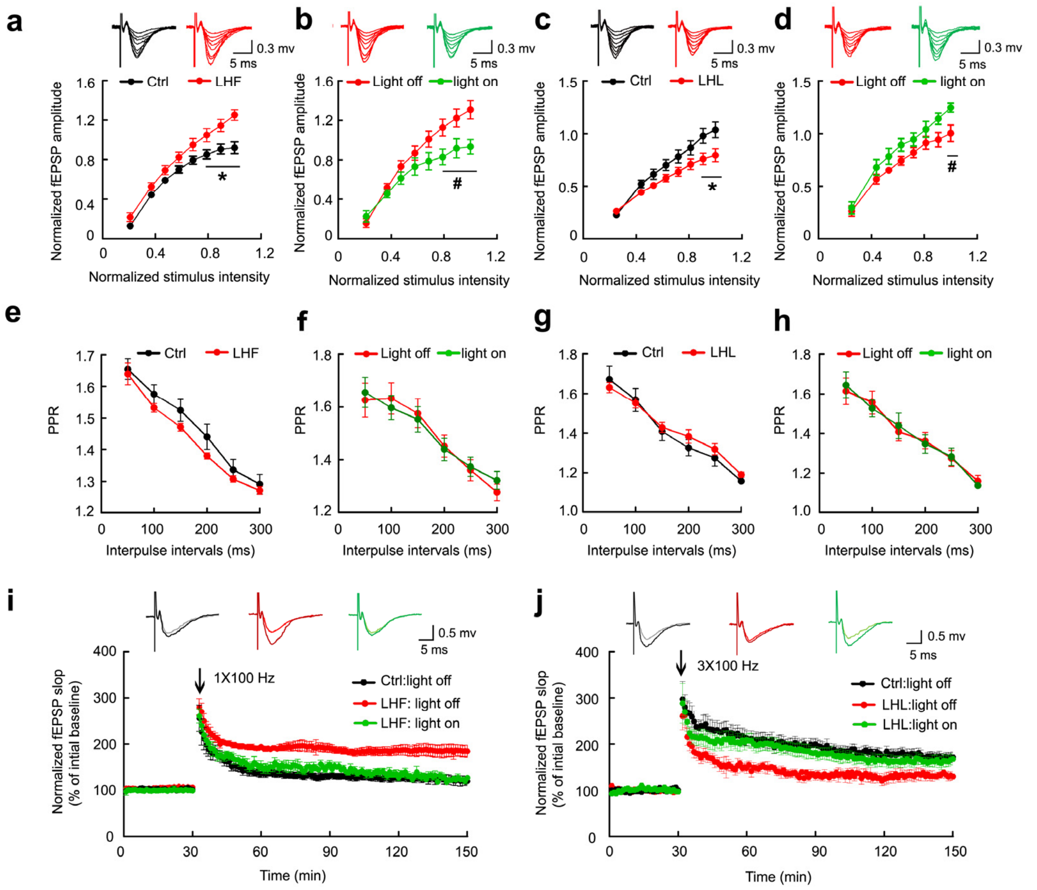

Supplementary Figure 6 LHF or LHL modulates postsynaptic plasticity *via* BLP–vCA1 inputs. (**a, b, i**) LHF increases input–output response and the slop of the evoked fEPSP in CA1, while blockage of BLP–vCA1 input by NpHR light on abolishes the increase. (**c, d, j**) LHL suppresses input–output response and slop of fEPSP, while simultaneous photostimulation of BLP–vCA1 by ChR2 light on rescues the suppression (n=10–11 slices from 6 mice each group, unpaired t test with Welch's correction for input–output response analyses, **P*<0.05 *versus* Ctrl; #*P*<0.05 *versus* light off). (**e**–**h**) LHF or LHL or opto–manipulation does not affect presynaptic release. Foot–shocks with avoidance training for 6 days or with simultaneous NpHR light on to block BLP–vCA1 input did not change paired–pulse ratio (PPR) in CA1(**e**,**f**). Simply foot–shocks or with simultaneous photostimulation of BLP–vCA1 input did not change PPR (**g**,**h**). Data were presented as mean ±s.e.m. n=10–11 hippocampal slices from 6 mice in each group, unpaired t test with Welch's correction.

Supplementary Figure 7

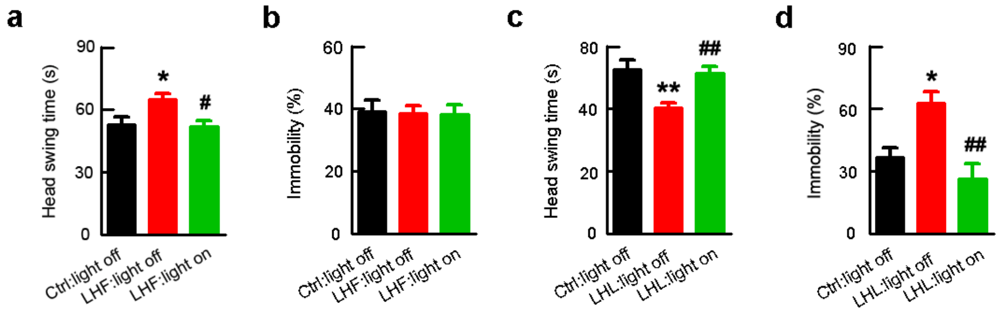

Supplementary Figure 7 Photomanipulation of BLP–vCA1 input modulates motivation states of LHL and LHF mice in FS. (**a**, **b**) Simultaneous blockage of BLP–vCA1 input by NpHR light on abolishes the LHF–increased head–shake responses without changing immobility time in FS. (**c**, **d**) Photostimulation of BLP–vCA1 by ChR2 light on improves the LHL–induced less head swings and immobility in FS (n= 7–8 mice per group, One–way ANOVA, Tukey's multiple comparisons test). Data were presented as mean ±s.e.m. **P*<0.05, ***P*<0.01 *versus* Ctrl:light off; #*P*<0.05, ##*P*<0.01 *versus* LHF:light off or LHL:light off.

Supplementary Figure 8

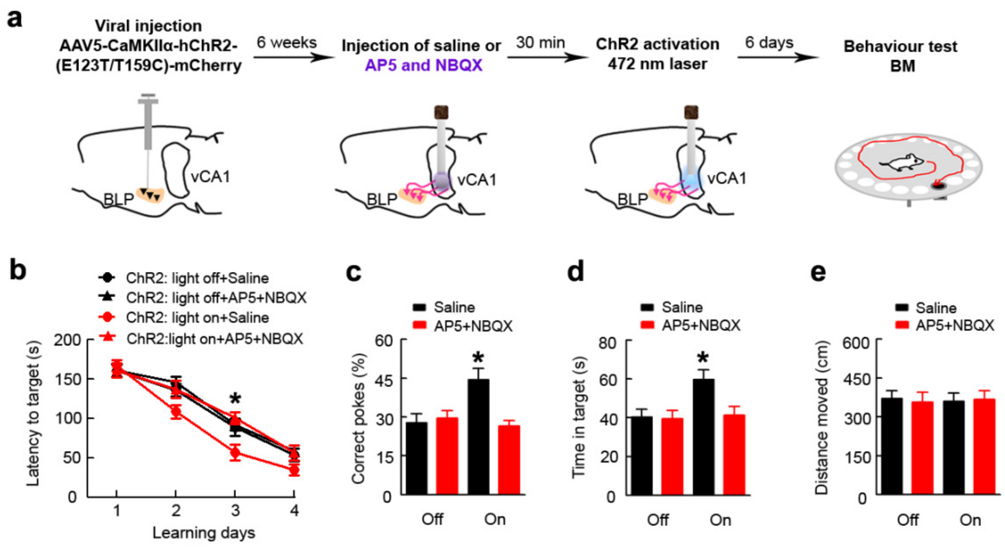

Supplementary Figure 8 Monosynaptic and glutamatergic BLP inputs to the vCA1 are sufficient to mediate potentiation on spatial memory. (**a**) Experiments were performed 6–7 weeks after AAV5–CaMKIIα–hChR2–(E123T/T159C)–mCherry injections in the BLP. Thirty minutes before laser stimulation, glutamate receptor antagonists (AP5+NBQX) or saline were bilaterally infused locally into the vCA1 using the same guide cannula to be used for light delivery *via* an optical fiber. (**b**–**d**) Bilateral AP5+NBQX injection in the vCA1 significantly attenuated light–induced enhancement in spatial learning (**b**) and memory (**c**,**d**) in the BM relative to saline trials. (**e**) Motor function was not affected (Ctrl, n=8, LHL, n=8, LHF, n=8, One–way ANOVA, Tukey's multiple comparisons test). Data were presented as mean ±s.e.m. *P<0.05 *versus* ChR2: light on +AP5+NBQX.

Supplementary Figure 9

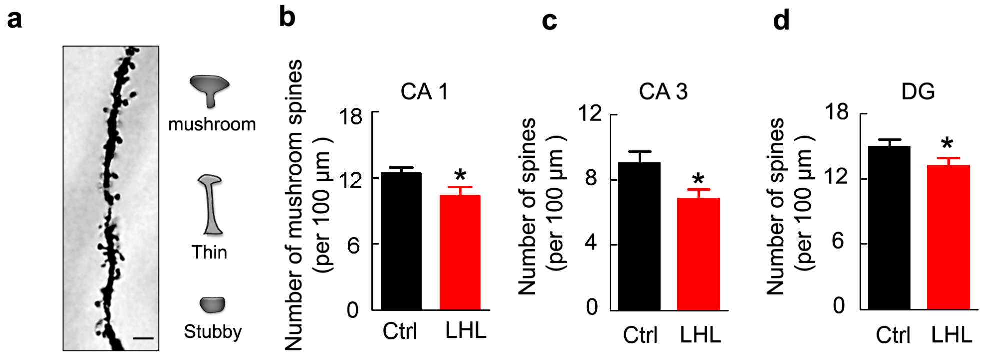

Supplementary Figure 9 LHL decreases spine generation in rats. The rats were treated as indicated in **Fig.1a**. (**a**) Representative images for Golgi staining of the dendritic spines in CA1 neurons of rats (scale bar=2.5 μm). (**b–d**) Spine number decreased in CA1, CA3 and DG of the LHL rats. Data were presented as mean ±s.e.m. At least 10 neurons from 7 rats were analyzed in each group, unpaired t test with Welch's correction, * *P*<0.05,* *P*<0.05 *versus* Ctrl.

Supplementary Figure 10

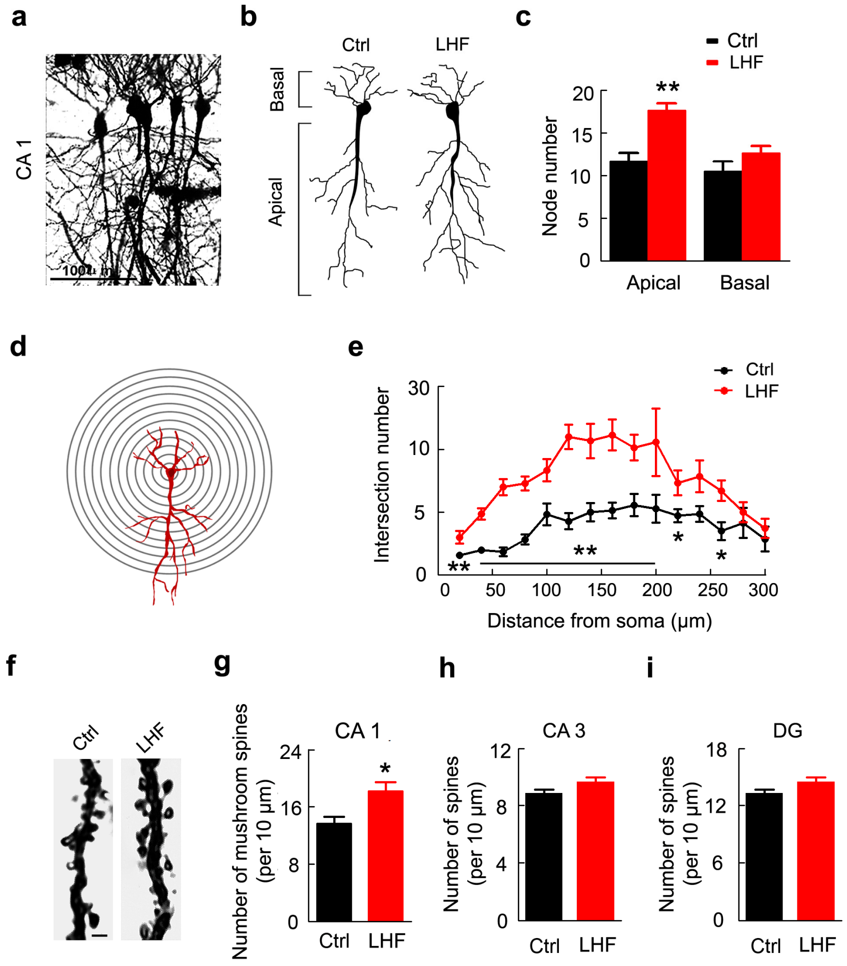

Supplementary Figure 10 LHF increases dendrite arborization and spine generation in rats. The rats were treated as indicated in **Fig. 1a**. (**a**) Representative images for Golgi staining of apical and basal dendrites in hippocampal CA1 (scale bar=100 μm). (**b**) Graphical draws show apical and basal dendrites in CA1 of Ctrl and LHF rats. (**c**) Quantitative analyses of the increased apical but not basal dendrite arborization in LHF group. (**d**, **e**)Increases of apical dendrites in the LHF analyzed by Sholl. (**f**) Representative images for Golgi staining of the dendritic spines in CA1 neurons of Ctrl and LHF rats (scale bar=1 μm). (**g–i**) Spine number increased in CA1 but not CA3 and DG of the LHF rats. Data were presented as mean ±s.e.m. At least 10 neurons from 6–7 rats were analyzed in each group, unpaired t test with Welch's correction, * *P*<0.05,** *P*<0.01 *versus* Ctrl.

Supplementary Figure 11

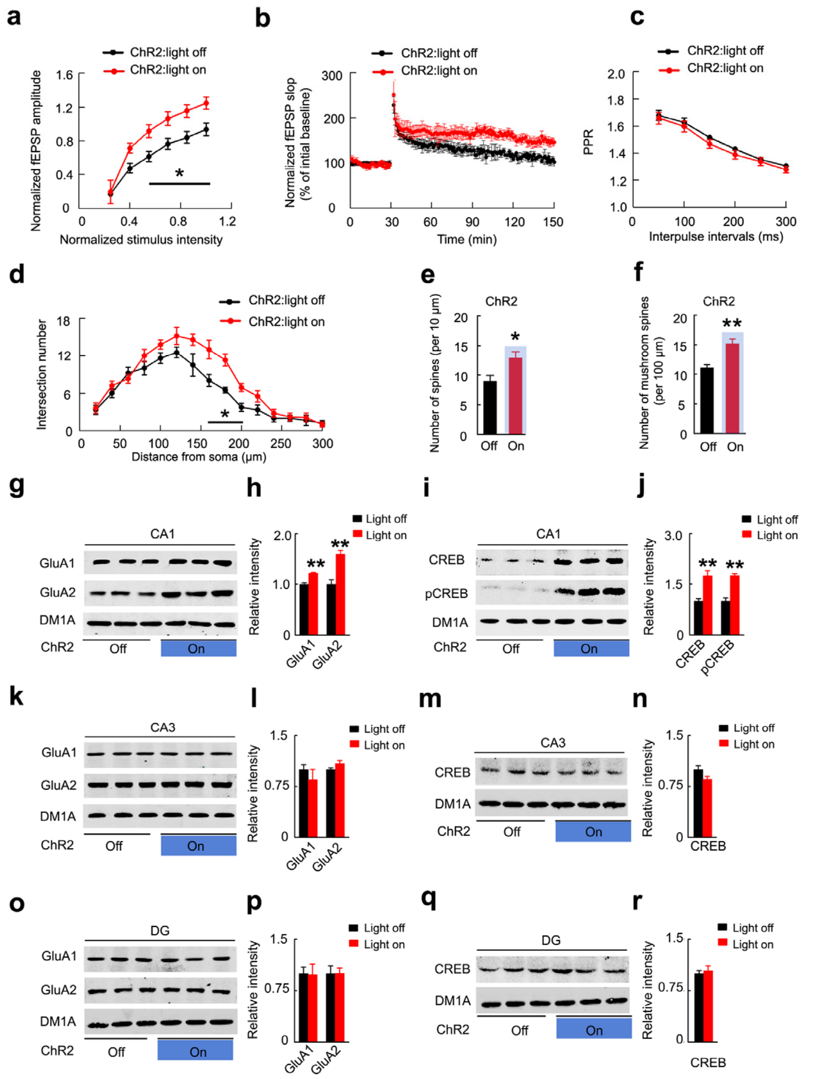

Supplementary Figure 11 Photostimulation of BLP–vCA1 facilitates synaptic plasticity in naïve mice. The mice were treated as indicated in **Fig. 6m**. (**a–c**) Photostimulation of BLP–vCA1 inputs facilitate basal synaptic transmission (**a**) and potentiate LTP (**b**) with no effect on PPR (**c**). (**d–f**) Photostimulation of BLP–vCA1 reinforces dendrite complexity shown by the increased numbers of intersection (**d**), spine (**e**) and mushroom–like spines (**f**). n=10 neurons from 6–7 mice per group. (**g–r**) Photostimulation of BLP–vCA1 increases levels of synaptic GluA1/2 (P2 fraction of CA1), and total CREB and the pCREB of CA1 extracts (**g–j**), while the levels of GluA1/2 and CREB in CA3 (**k–n**) and DG (**o–r**) were not changed. n=3 per group. Data were presented as mean±s.e.m., unpaired t test with Welch's correction, * $P<0.05$, ** $P<0.01$ *versus* light off.

Supplementary Figure 12 Full version of Western blots in Figure 7

e

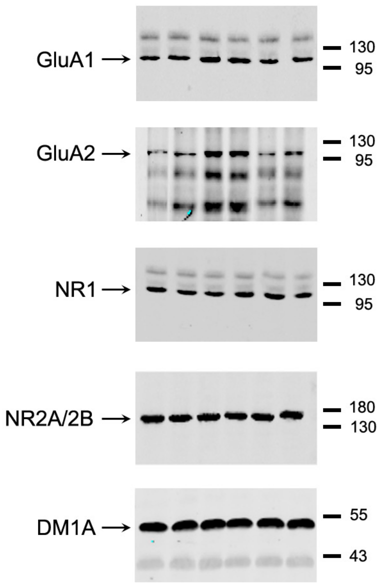

g

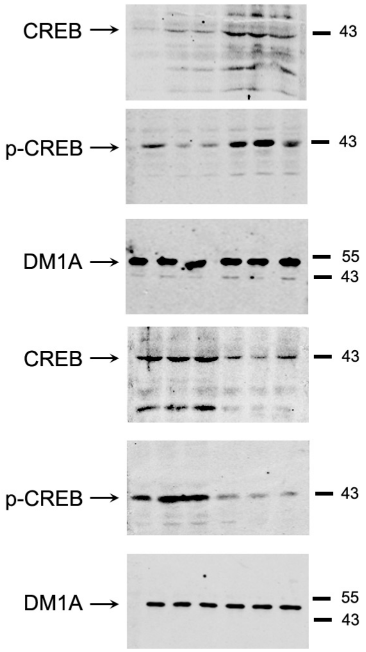

i

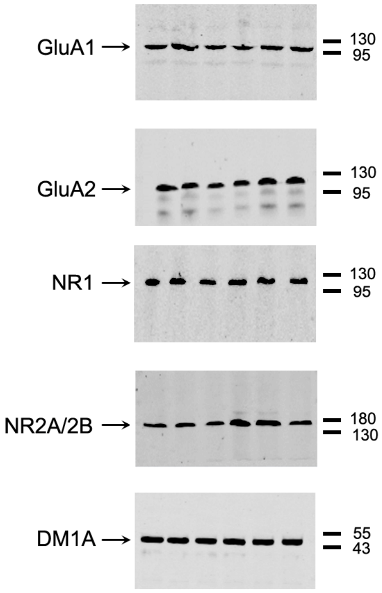

k

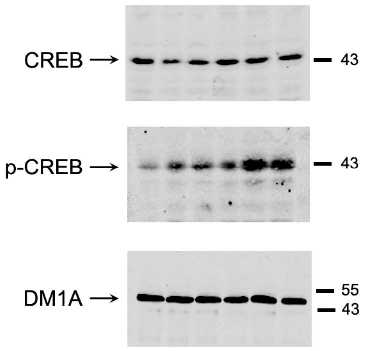

Supplementary Figure 13 Full version of Western blots in Supplementary Figure 11

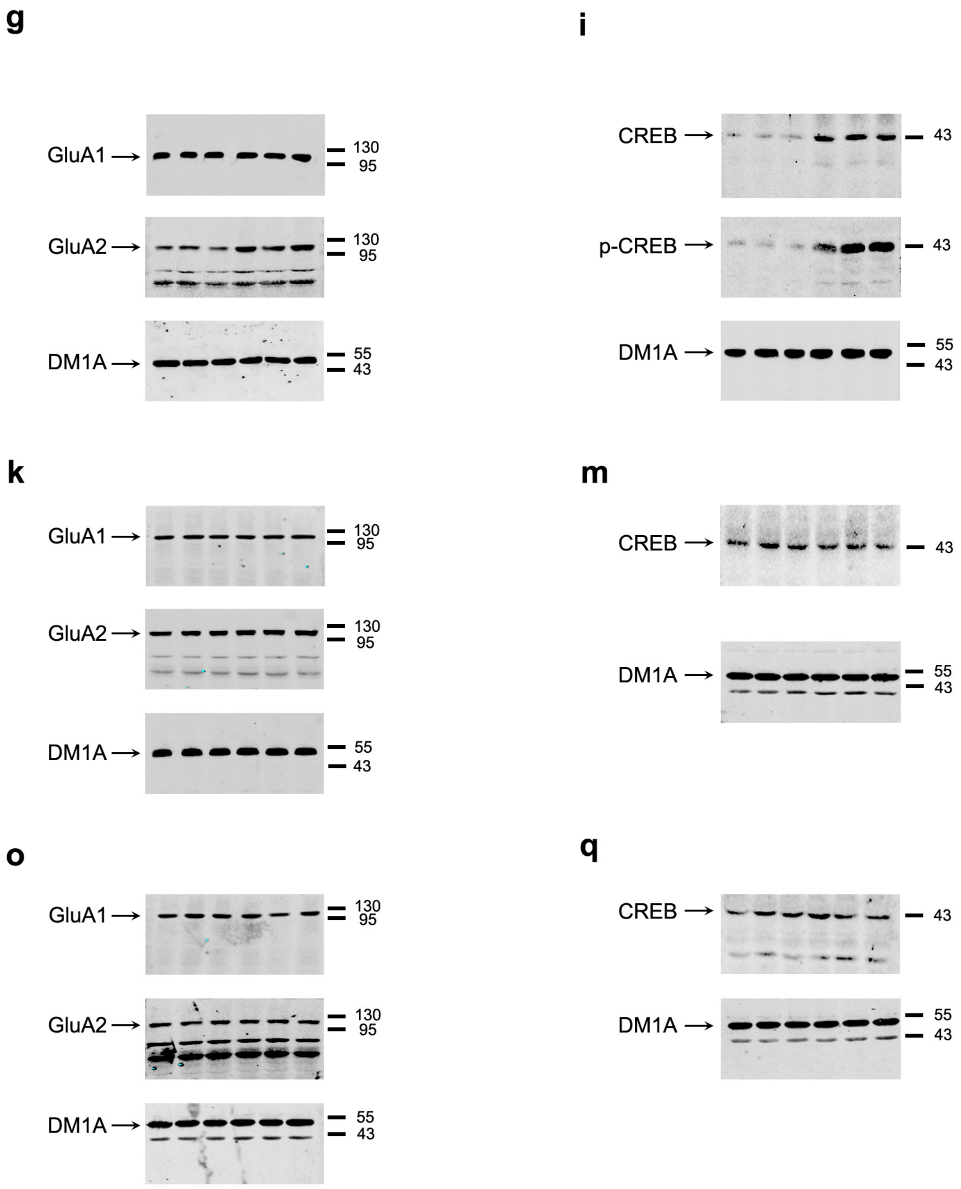

Supplement: Supplementary Information — Supplementary Figures 1-13 [file ncomms11935-s1.pdf]
